# Protein Kinase C Delta Negatively Modulates Canonical Wnt Pathway and Cell Proliferation in Colon Tumor Cell Lines

**DOI:** 10.1371/journal.pone.0058540

**Published:** 2013-03-08

**Authors:** José G. Hernández-Maqueda, Luis Bernardo Luna-Ulloa, Paula Santoyo-Ramos, M. Cristina Castañeda-Patlán, Martha Robles-Flores

**Affiliations:** Department of Biochemistry, Faculty of Medicine, Universidad Nacional Autónoma de México (UNAM), Mexico City, Mexico; University of Munich, Germany

## Abstract

The tumor suppressor Adenomatous Polyposis coli (*APC*) gene is mutated or lost in most colon cancers. Alterations in Protein kinase C (PKC) isozyme expression and aberrant regulation also comprise early events in intestinal carcinomas. Here we show that PKCδ expression levels are decreased in colon tumor cell lines with respect to non-malignant cells. Reciprocal co-immunoprecipitation and immunofluorescence studies revealed that PKCδ interacts specifically with both full-length (from non-malignant cells) and truncated APC protein (from cancerous cells) at the cytoplasm and at the cell nucleus. Selective inhibition of PKCδ in cancer SW480 cells, which do not possess a functional β-catenin destruction complex, did not affect β-catenin-mediated transcriptional activity. However, in human colon carcinoma RKO cells, which have a normal β-catenin destruction complex, negatively affected β-catenin-mediated transcriptional activity, cell proliferation, and the expression of Wnt target genes *C-MYC* and *CYCLIN D1*. These negative effects were confirmed by siRNA-mediated knockdown of PKCδ and by the expression of a dominant negative form of PKCδ in RKO cells. Remarkably, the PKCδ stably depleted cells exhibited augmented tumorigenic activity in grafted mice. We show that PKCδ functions in a mechanism that involves regulation of β-catenin degradation, because PKCδ inhibition induces β-catenin stabilization at the cytoplasm and its nuclear presence at the *C-MYC* enhancer even without Wnt3a stimulation. In addition, expression of a dominant form of PKCδ diminished APC phosphorylation in intact cells, suggesting that PKCδ may modulate canonical Wnt activation negatively through APC phosphorylation.

## Introduction

Colorectal cancer (CRC) is one of the most prevalent cancers and is a leading cause of cancer mortality worldwide. Current evidence indicates that the Wnt cascade is the dominant force in controlling intestinal epithelium homeostasis and stem cell maintenance [1]. Adenomatous polyposis coli (*APC*), originally identified as an intestinal tumor suppressor gene, was recognized as a key negative regulator of the cascade. A great amount of experimental evidence has shown that *APC* is the gatekeeper in the molecular pathogenesis of the majority of sporadic and hereditary forms of colorectal carcinoma [1], [2]. In the adenoma-carcinoma sequence of sporadic colorectal carcinoma (CRC), the smallest identifiable lesion is an aberrant crypt focus (ACF) and two types of ACFs have been distinguished. The most common is associated with a hypercellular or hyperplastic crypt that seldom develops into malignant carcinomas. The second type, the dysplastic ACF or unicryptal adenoma, occurs frequently in carcinoma-associated colon mucosa. Most of these dysplastic ACFs bear *APC* mutations, whereas nonmalignant hyperplastic ACFs may arise from activating mutations in *K-RAS* or complementary mutations in the upstream component *B-RAF* [3], [4].

Canonical Wnt signaling operates through regulating the phosphorylation and degradation of the transcription co-activator β-catenin. Without stimulation by Wnt, β-catenin is assembled into the so-called destruction complex, in which APC protein plays a central role, and includes Axin, GSK3β, and Casein kinase 1 (CK1). This complex directs a series of phosphorylation events in β-catenin that targets it for ubiquitination and subsequent proteolysis via the proteosome [5], [6]. Stimulation by Wnt leads to inhibition of β-catenin breakdown, allowing β-catenin to accumulate, enter the nucleus, and activate a Wnt target gene program [5], [7]. However, despite this outline of canonical signaling that has been established as result of intense work, major questions remain on how the ß-catenin destruction complex operates and what role APC plays.

In addition, although the participation of APC in the regulation of ß-catenin stability is critical for Wnt signaling, some data support additional roles for APC in Wnt regulation. APC contains nuclear localization and export signals that allow it to shuttle in and out of the nucleus and to regulate ß-catenin nuclear export [8]. Furthermore, APC coordinates the cyclic exchange of Wnt co-regulator complexes in the DNA, thus acting directly on promoters in transcriptional repression [9].

Experimental evidence has shown that the process of intestinal carcinogenesis is also associated with alterations in the activity and expression of Protein kinase C (PKC) isoforms [10], [11]. Intestinal epithelial cells express at least nine PKC isoforms, which include PKCα, PKCβI, PKCβII, PKCδ, PKCε, PKCθ, PKCη, PKCζ and PKC-λ/ι [12], suggesting that this family plays important roles in the maintenance of intestinal epithelium homeostasis. The experimental evidence is consistent with the notion that in the intestinal epithelium, some PKC isoforms, such as PKCβII, -ε, and the atypical PKCs, preferentially function to promote cell proliferation and survival, while others, such as PKCα and PKCδ, behave as tumor suppressors [13], [14]. In the case of PKCδ, and consistent with a potential tumor suppressor role, some studies have reported that several proto-oncogenes transform cells, at least in part, by causing the loss of PKCδ activity [15]. Conversely, overexpression of PKCδ inhibited the transformed phenotype of Src overexpression in rat colonic epithelial cells, causing an arrest in cellular proliferation [16]. In addition, it has been reported that PKC delta is an important regulator of apoptosis [15], [17].

Although many studies have implicated PKC isoforms in the Wnt signaling pathway, the molecular mechanisms involved in their crosstalk in cancer initiation and progression remain poorly understood. We have recently reported that atypical PKCζ positively modulates canonical Wnt signaling by controlling nuclear β-catenin localization [18]. Unexpectedly, we have also found that PKCδ interacts with APC *in vivo* in the cytoplasm and at the cell nucleus with PKCδ in both normal and malignant colon cell lines. Here we show that PKCδ negatively modulates canonical Wnt signaling participating in the regulation of β-catenin stability. Our data suggest that this occurs by means of PKCδ-mediated phosphorylation of APC.

## Materials and Methods

### Reagents and Antibodies

Isozyme-specific polyclonal antibody against the C-terminus of PKCδ (C-17, sc-213) and the APC antibody (sc-53165) were obtained from Santa Cruz Biotechnology Inc. (Sta. Cruz, CA, USA). Antibodies against β catenin (E-5, sc-7963) and anti-TCF4 (H-125, sc-13027) were also obtainesd from Sta. Cruz Biotechnology. Anti β-tubulin antibody was purchased from Zymed (cat. 18-0093). Phospho-(Ser) PKC substrate antibody was obtained from Cell Signaling. Goat anti-mouse and anti-rabbit IgG-horseradish peroxidase-conjugates were from Pierce (Rockford, IL, USA). PKCδ- selective inhibitor rottlerin, GSK-3 Inhibitor IX (BIO, (2′Z, 3′E)-6-Bromoindirubin-3′ –oxime) and Protein A-sepharose were obtained from Calbiochem/Merck (Darmstadt, Germany). Nuclei isolation kit was purchased from Sigma (St. Louis MO, USA). RNA was reverse transcribed using SuperScript One-Step RT-PCR with Platinum Taq (Invitrogen). All other chemicals were reagent grade.

### Ethics Statement

All animals were handled in strict accordance with good animal practice as defined by the Animal Experimental Bio-Ethics Guidelines of the Instituto Nacional de Ciencias Médicas y Nutrición Salvador Zubirán, Mexico. In addition, all work with animals was approved by the Animal Experimental Bio-Ethics Committee of the Faculty of Medicine, Universidad Nacional Autónoma de México. When indicated, mice were euthanized by CO_2._

#### Plasmids

The pTOPFlash and pFOPFlash reporter plasmids were obtained from Upstate Biotechnology. The plasmid encoding dominant-negative PKCδ (PKCdelta K376R-HA, Addgene plasmid 10819) [19] was obtained from Addgene (Cambridge, MA, USA), a non-profit organization dedicated to making it easier for scientists to share plasmids. For knockdown PKCδ experiments, we used the pSUPER.PKCdelta.RNAi plasmid donated by Dr. Alex Toker to Addgene (Addgene plasmid 10819) whose construction and effectiveness are described in [20]. The plasmid encoding wild-type PKCδ was a generous gift from Drs. Jae-Won Soh and Kevin Catt at the Endocrinology and Reproduction Research Branch, NICHD, NIH, USA.

### Cell Culture

RKO (human colon carcinoma), HCT116 (human colorectal carcinoma), HT29 or SW480 (human colorectal adenocarcinoma) malignant cells and non-malignant IEC-18 (non-transformed rat epithelial intestinal crypt cells) and 112CoN (human colon) cells were all obtained from the American Type Culture Collection (Manassas, VA, USA). RKO and 112CoN cells were maintained in Dulbecco’s modified Eagle’s medium (DMEM) supplemented with 10% fetal bovine serum (FBS), antibiotics (120 mg/ml penicillin and 200 mg/ml streptomycin) and 2 mM L-glutamine. IEC-18 cells were cultured in the same medium but were supplemented with 5% FBS, antibiotics, L-glutamine, 4.5 g/L glucose and 0.1 units/mL insulin. HT-29 and HCT116 cells were maintained in McKoy medium supplemented with 10% FBS, antibiotics, and 2 mM glutamine. SW480 cells were maintained in DMEM F-12 supplemented with 5% FBS, antibiotics and 2 mM glutamine. All cells were cultured in a humidified 5% CO_2_ incubator at 37°C.

The cell lines used in this study were authenticated by DNA profiling using short tandem repeat (STR) analysis on a AmpFlSTR® Identifier™ PCR Amplification System at “Instituto Nacional de Medicina Genómica” (INMEGEN), Mexico, D.F.

### Transfection and Luciferase Reporter Gene Assay

Transfection was carried out with Lipofectamine 2000 (Invitrogen) or with FuGENE 6 (Roche-Applied-Science) according to the manufacturer’s instructions. Briefly, cells were seeded in 24-well plates at a density of 1×10^3^ cells/well (RKO) or 1×10^4^ cells/well (SW-480). Twenty-four hours after seeding, cells were placed in a serum-free medium and transiently transfected with 0.7 µg of reporter plasmid (pTOPFlash) or control plasmid (pFOPFlash) plus 0.05 µg of pRL luciferase plasmid (transfection control). Luciferase reporter activity was measured 24 hours after transfection in cell lysates using the Dual luciferase assay kit (Promega; Madison, WI, USA). Activity was normalized with respect to the activity of *Renilla luciferase* or with the protein content in each sample. To express a dominant-negative form (kinase-inactive K376R) of PKCδ, cells were transiently transfected with 1 µg of plasmid PKCdelta.K376R obtained from Addgene (ID16389) using Lipofectamine 2000.

### Incubation with Wnt3a Ligand and Pharmacological Inhibition of PKC Delta

At 24-h post-transfection with reporter plasmids, RKO cells were serum-starved (2% instead of 10%) for 8 h and treated with Wnt3a (50 ng/ml or 100 ng/ml; R&D Systems, Minneapolis MN, USA) for a total 12-h period. During the last 3 h, cells were also incubated in the absence or presence of 6 µM of the PKC delta inhibitor rottlerin. Different concentrations of rottlerin were employed (3, 4, 6 and 10 µM) in dose-response assays. After the total incubation period, cells were washed and lysed for luciferase activity determination.

### PKCδ Knockdown

In the cases of transient silencing of PKCδ, cells were transfected with 1 µg of pSuper.PKCdelta.RNAi (constructed and probed by Dr. Alex Toker, as described in [20], or with control plasmid obtained from Addgene (ID 10803), using Lipofectamine 2000. In some experiments, cells were also transiently co-transfected with the pTOPFlash or pFOPFlash plasmids and with increasing amounts of pSuper.PKCδ.RNAi plasmid, maintaining constant the total amount of transfected DNA (2 µg) constant by adding pSuper plasmid control.

To generate stable transfections, RKO cells were transfected with either 1 µg of control pSuper plasmid or co-transfected with 900 ng of pSuperPKCδ.RNAi plus 100 ng of pSUPERpuro plasmids. Stable transfectants were selected with 3 µg/ml puromycin (Sigma) for 4 weeks, and clones were picked and screened for PKCδ silencing by flow cytometry.

### Western Blot Analysis

Cells were homogenized in ice-cold buffer (20 mM Tris-HCl, pH 7.5, 10 mM EGTA, 2 mM EDTA, 1% Triton X-100, 1 mM Phenylmethylsulfonyl fluoride, 10 µg/ml leupeptin, and 0.1 mg/ml trypsin inhibitor). Samples of protein (50−100 µg) were separated by 4−12%-gradient SDS-PAGE (Invitrogen) followed by their electrophoretic transfer onto nitrocellulose membranes (Bio-Rad). The membranes were blocked with 5% non-fat dry milk and incubated overnight at 4°C with the corresponding primary antibody. Detection was achieved using the Supersignal kit (Pierce; Rockford, IL, USA) with a horseradish peroxidase-conjugated second antibody. An actin antibody was employed as a control for equal loading.

### Immunofluorescence Analysis

HT-29 and IEC-18 cells were grown on coverslips. Cells were washed with PBS, fixed with methanol at −20°C during 10 min, permeabilized with 0.3% Triton X-100, and blocked with 4% IgG-free BSA (Research Organics; Cleveland, OH, USA) for 1 h. RKO or SW480 cells grown on coverslips were washed with PBS, fixed in ice-cold acetone for 5 min, washed in PBS, and blocked with 1% IgG-free BSA for 1 h. All cells were incubated overnight at 4°C with the corresponding primary antibodies in blocking solution, washed three times with PBS, and incubated for 1 h in darkness at room temperature with secondary antibodies (FITC-conjugated goat anti-rabbit and TRITC-conjugated goat anti-mouse). After washing, the coverslips were mounted with the antifade reagent Vectashield. Cell fluorescence was examined using a confocal microscope (Leica TCS SP5) with a krypton argon laser.

### RT-PCR

Total RNA was isolated from cells using Trizol reagent (Life Technologies). Total RNA was reverse transcribed using SuperScript One-Step RT-PCR with Platinum Taq (Invitrogen) using the following gene-specific primers: human *C-MYC* Fw: 5′ TACCCTCTCAACGACAGCAG; human *C-MYC* rev: 5′ TCTTGACATTCTCCTCGGTG. *GAPDH* was reverse transcribed under the same conditions to be used as a control, with the following primers: Fw 5′ CATCTCTGCCCCCTCTGCTGA; Rev 5′GGATGACCTTGCCCACAGCCT. For human *CYCLIN D1* the primers used were: Fw: 5′ AGCTCCTGTGCTGCGAAGTGGAAAC-3′ Rev 5′ AGTGTTCAATGAAATCGTGCGGGGT-3′.

### Immunoprecipitation

Cells were washed and homogenized in ice-cold lysis buffer containing 50 mM Tris, 150 mM NaCl, 0.5% Triton X-100 at pH 7.5 and a mixture of protease inhibitors and protein phosphatase inhibitors. The protein concentration in the supernatant was measured utilizing a detergent-compatible protein assay (Bio-Rad). Aliquots of these extracts (1 mg/ml) were incubated overnight at 4°C with 2 µg/ml of primary antibody with gentle shaking. Then, 25 µl protein A-sepharose (30%, Calbiochem) was added and incubation continued for 2 h. Immune complexes were then washed twice with buffer A (50 mM Tris-HCl, 0.6M NaCl, pH 8.3) supplemented with 0.1 mg/ml trypsin inhibitor and 1 mM PMSF, and once with buffer B (50 mM Tris HCl pH 7.5, 0.15 M NaCl) containing protease and phosphatase inhibitors.

### Proliferation (Viability) Assay

The presence of metabolically active viable cells was used as an index of cell proliferation using the 3-(4,5-dimethylthiazole-2-yl)-2,5-biphenyl tetrazolium bromide (MTT) assay. RKO cells were grown in 24-well plates at 1.6×10^5^ cells/well for 24 hrs. After this period, cells were serum-starved (2% instead of 10%) for a 6 h period; subsequently, recombinant mouse Wnt3a (50 ng/ml) was added to the medium to stimulate cells for a total 24-h period in the absence or presence of 6 µM of the PKC delta inhibitor rottlerin. Cells were incubated with MTT (0.5 mg/mL) for an additional 3 h at 37°C. The reaction was stopped adding 1 ml of isopropanol acid to each well. Formazan salts were dissolved and quantified spectrophotometrically at 570 nm.

### Xenograft Tumor Model

Stably transfected RKO cells with control pSUPER plasmid (thus expressing endogenous wild type PKCδ) or with pSuperPKCδ-RNAi (PKCδ knock-down) were collected by trypsinization. Then, 1 × 10^6^ cells per injection site were resuspended in high concentration Matrigel (BD Biosciences), diluted in PBS to 50% final concentration and subcutaneously injected into the flanks of 4-week-old CD1 nude mice. Each mouse was injected at 2 sites, at the right flank with control cells, and at the left flank with knockdown PKCδ cells. Five weeks after inoculation, animals were euthanized and tumors were removed and weighted.

### Chromatin Immunoprecipitation Experiments

DNA-protein cross-linking was performed in RKO cells grown in the absence or presence of Wnt3a conditioned medium and in the absence or presence of rottlerin (6 µM) with 1.1% formaldehyde for 20 min. Then, 0.125 M glycine was added and cells were washed with PBS. Nuclei were purified from cell samples using a Nuclei Isolation Kit (Sigma-Aldrich) according to manufacturer’s instructions. The nuclei were lysed in buffer (1 mM EDTA, 0.5 mM EGTA and 10 mM Tris-HCl pH 8.0) supplemented with protease and phosphatase inhibitors. After this, the samples were sonicated to fragment chromatin (10 for five, 20-sec pulses) to an average length of approximately 1,000 bp followed by centrifugation. The chromatin solution was pre-cleared with the addition of protein A-sepharose (previously blocked with sheared herring sperm DNA and bovine serum albumin) overnight at 4°C. The supernatant was incubated with specific antibodies overnight and then with protein G-Sepharose beads for 1 h. After an extensive wash step, the complexes were eluted with buffer (100 mM NaHCO_3_ and 1% SDS) for 15 min followed by centrifugation. The supernatant was recovered and incubated with RNase at 37°C for 30 minutes. Afterward, Proteinase K was added and incubation was continued overnight at 55°C. Then, NaCl 5M was added and incubation continued for 4 h at 65°C. Finally, the DNA was extracted, purified, and utilized for PCR. Primers for the human *C-MYC* enhancer monitored the third *TCF/LEF-1* site (spanning from −1447 to −1144 relative to the P1 RNA start site +1). These primers were used and validated as described in (9) and were the following: forward 5′ GTGAATACACGTTTGCGGGTTAC3′, and reverse 5′AGAGACCCTTGTGAAAAAAACCG 3′. The primers employed corresponded to a negative control sequence 1 kb upstream of *C-MYC* promoter and were the following: forward 5′ GCAGCAAAATCCAGCATAGCG 3′, and reverse 5′ CCACCACCTCCAAAAGAGAAAAC 3′. PCR of the input DNA prior to immunoprecipitation was utilized as a control.

### Metabolic Labeling and Determination of Specific Phosphorylation

RKO cells were transfected with the pHACE (void) or pDN-PKCδ plasmids. 24 h post-transfection RKO cells were serum-starved (2% instead of 10%) for 4 h, washed and incubated with 150 µCi/ml [^32^P] Pi in phosphate-free DMEM medium supplemented with 2% FBS for a total period of 4 h. During the last hour, cells were also incubated in the absence or presence of Wnt3a (50 ng/ml). Cell lysates were prepared and APC was immunoprecipitated with specific antibody (2 µg/ml) as previously described, subjected to SDS-PAGE, transferred to nitrocellulose, and processed for autoradiography or for Western blotting. Phosphorylated APC was quantified by densitometric scanning. Total protein was also determined in the exposed gel by densitometric scanning, and total specific phosphorylation was calculated as the ratio of ^32^P-labeled APC to total protein.

### Statistical Analysis

Data are expressed as mean ± Standard error of the mean (SEM). Statistical analysis of the data was performed by the Student’s *t-*test. A value of P<0.05 was considered statistically significant.

## Results

### PKCδ and APC Co-immunoprecipitate in a Reciprocal Manner and Co-localize in Both Normal and Malignant Cells

**Figure 1:**
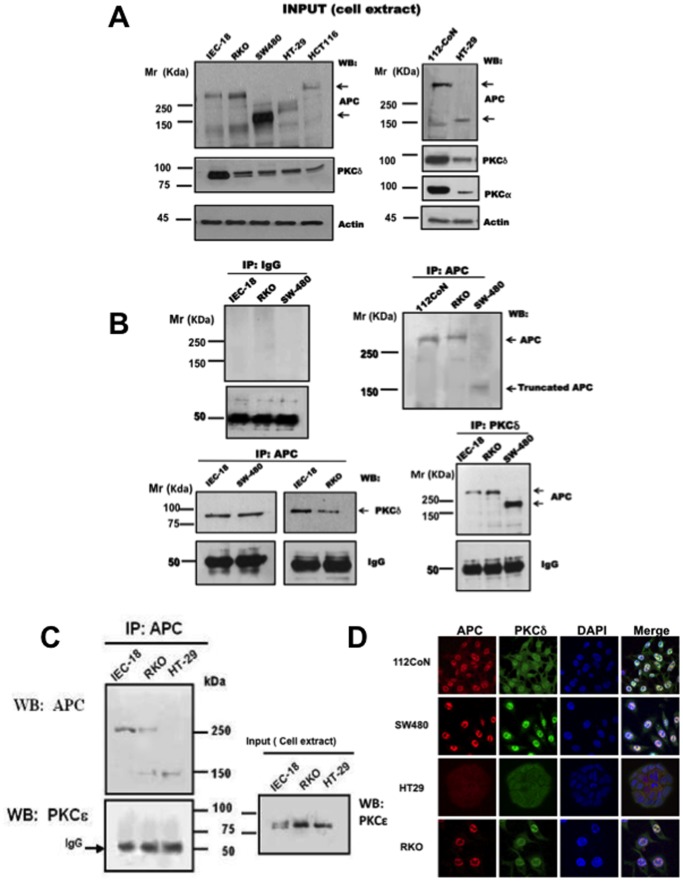
APC protein specifically interacts with PKCδ. **A)** Expression levels of APC protein and PKCδ in colon cell lines. Cell extracts from colon cell lines were prepared as described in Methods and used as starting material for immunoprecipitation studies. **B) Upper panel:** Immunoprecipitation controls are shown: IgG was used as negative control, and the analysis of APC immuprecipitate with APC antibody showing that immunoprecipitation was successful was used as positive control. **Lower panel:** APC was immunoprecipitated using an antibody directed to its N-terminal region or using antibody against PKCδ, from total cell extracts obtained from IEC-18 non malignant cells, or from total cell extracts obtained from RKO or SW480 malignant cells. Immunoprecipitates were analyzed by Western blot for the presence of PKC isoforms or APC as indicated in the Figure. The results are representative of three independent experiments using different cell preparations. **C)** APC was immunoprecipitated using an antibody directed to its N-terminal region or using antibody against PKCε, from total cell extracts obtained from IEC-18 non malignant cells, or from total cell extracts obtained from RKO or HY-29 malignant cells. Immunoprecipitates were analyzed by Western blot for the presence of PKCε or APC as indicated in the Figure. The results are representative of three independent experiments using different cell preparations. **D)** APC co-localizes with PKCδ in both normal and malignant cells. Cells were fixed, permeabilized and coimmunostained with antibodies against APC and PKCδ. Fluorescence was analyzed by laser confocal microscopy as described under “Materials and Methods”. PKCδ was visualized with FITC-conjugated goat anti-rabbit antibody and APC with TRITC-conjugated goat anti-mouse antibody. Data are representative of four independent experiments.

To investigate whether there are interactions between PKC isozymes and the APC suppressor protein, co-immunoprecipitation studies were performed in the following several human intestinal cell lines that reflects differences in Wnt signaling genetic context with respect to APC expression: 112CoN (human) and IEC-18 (rat) are non-malignant cell lines that express the normal APC suppressor protein (Figure 1A); RKO human cells are malignant but express wild- type APC. It is important to mention that these cells have been largely considered to feature normal Wnt signaling. However, it has been reported that they have mutated the Wnt antagonist Naked cuticle 1 (*NKD1*) which negatively modulates Dvl levels [21]. Contrariwise, HT-29 and SW480 human malignant cells express truncated versions of APC, thus altering Wnt signaling (Figure 1A). Consistent with previous reports, it can also be observed that colon tumor cell lines exhibit reduced levels of PKCδ in comparison with non-malignant cells (Figure 1A). APC was immunoprecipitated from normal and from malignant cell extracts and the immunoprecipitates were analyzed by Western blot. As can be observed in Figure 1B, PKCδ co-immunoprecipitated in a reciprocal manner with APC from normal IEC-18 cells. Surprisingly, despite that PKCδ protein levels are low in malignant cells, this isozyme also co-immunoprecipitated reciprocally with truncated APC. The interaction is specific, because PKCε, which is overexpressed in cancerous cells in comparison with IEC-18 non malignant cells, did not co-immunoprecipitate with APC as shown in the Figure 1C. Consistent with these results, immunofluorescence assays followed by confocal microscopy analysis as presented in Figure 1D, showed co-localization of APC with PKCδ both in malignant and normal cells mainly at the nuclei.

### Pharmacological Inhibition of PKCδ Improved β-catenin Transcriptional Activity in RKO Cells Dose-dependently

**Figure 2:**
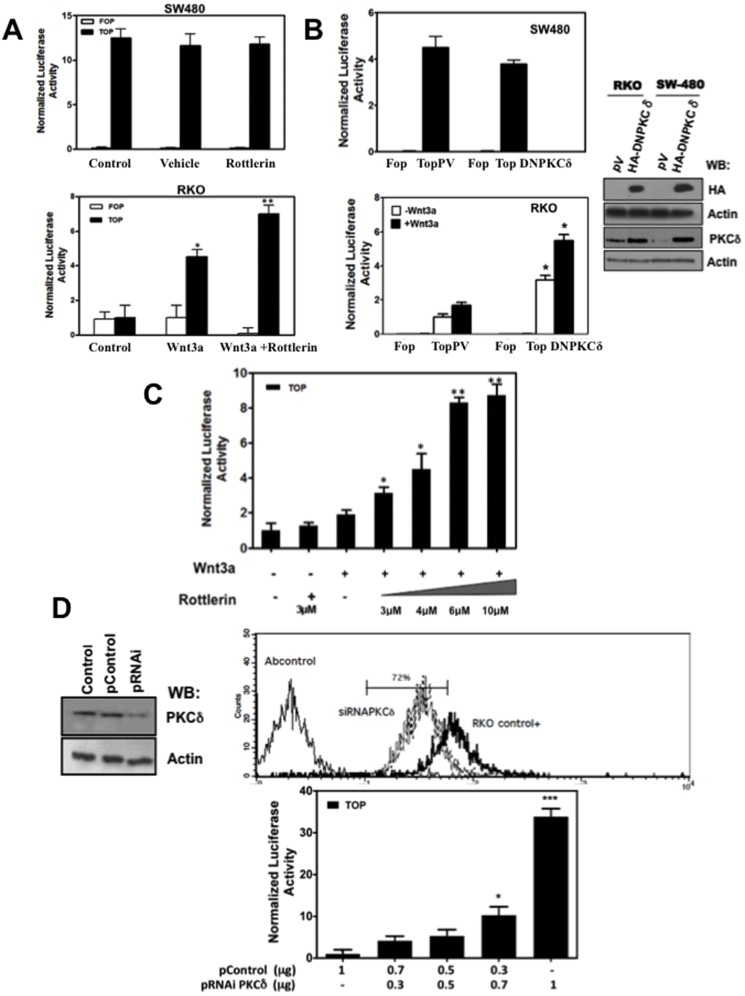
PKCδ inhibition increased β-catenin transcriptional activity in RKO (normal Wnt signaling) but not in SW480 (constitutively active Wnt signaling) tumoral cells. **A**) Pharmacological inhibition of PKCδ with rottlerin. SW480 (upper panel) and RKO cells (lower panel) were transfected with TOPFlash or FOPFlash reporter plasmid. 24 h post-transfection, SW480 cells were incubated in the absence or presence of 3 µM rottlerin for 3 h and then washed and lysed for luciferase activity determination. In the case of RKO cells, they were serum-starved for a period of 8 hours, and then recombinant mouse Wnt3a (100 ng/ml) was added to the medium to stimulate cells for a total 12 h period. During the last 3 hours, cells were also incubated in the absence or presence of 3 µM rottlerin. After this, cells were washed and lysed for luciferase activity determination. The activity was normalized with respect to the activity of Renilla luciferase or with respect to the protein content in each sample. The results represent the means ± S.E.M. of at least three independent experiments. *p<0.05, **p<0.01 **B**) Effect of expression of a dominant-negative form of PKCδ in the β-catenin transcriptional activity. SW480 or RKO cells were transiently co-transfected with void plasmid or with 1 µg of HA-tagged kinase-dead K376R PKCδ plasmid and with the TOPFlash/FOPFlash reporter plasmid. 24 h after transfection, luciferase activity was assayed and normalized with respect to Renilla luciferase activity. The HA-tagged kinase-dead PKCδ expression and endogenous PKCδ expression were verified in SW480 and RKO cells by Western blot 24 h after transfection as shown at the right of the figure. The results represent the means ± S.E.M. of at least three independent experiments. *p<0.05. **C**) PKCδ inhibition increase in a dose-dependent manner the β-catenin-mediated transcriptional activity in RKO cells. 24 h post-transfection with TOPFlash reporter plasmid, RKO cells were serum starved for 8 h and incubated in the absence or presence of 100 ng/ml Wnt3a for a total 12 h period. During the last 3 h, cells were also incubated in the absence or presence of increasing amounts of rottlerin as indicated in the figure. Then, cells were washed and luciferase activity was measured and normalized with respect to the total protein content in the cell lysate. The results represent the means ± S.E.M. of at least three independent experiments. *p<0.05, **p<0.01. **D**) PKCδ knockdown increased the β-catenin transcriptional activity in a dose dependent way without Wnt ligand stimulation. At the upper part of the figure, it is shown the efficiency of the knockdown of PKCδ determined by flow cytometry and by Western blot 24 h post-transfection with 1 µg of pSuperPKCδ.RNAi or with control plasmid. RKO cells were transiently co-transfected with the pTOPFlash plasmid and with increasing amounts of pSuper.PKCδ.RNAi plasmid maintaining constant the total amount of transfected DNA (1 µg) by adding pSuper plasmid control. 24 h post-transfection, luciferase activity was assayed and normalized with respect to the total protein content in the cell lysate. The results represent the means ± S.E.M. of at least three independent experiments. *p<0.05, ***p<0.001.

In order to examine the effect of PKCδ inhibition on canonical Wnt signaling, we employed the TOPFlash/FOPFlash β-catenin/TCF transcriptional activity reporter system. We used RKO human malignant cells which express wild-type APC protein and that are responsive to ligand in comparison with SW480 human malignant cells, which express a truncated version of APC and that have constitutively active Wnt signaling. The specificity of the PKCδ inhibitor, rottlerin, was examined by *in vitro* activity assays and also the dose (3 µM) required to effectively inhibit PKCδ (Figure S1). At 24 h post-transfection with the pTOPFlash or pFOPFlash plasmids, RKO malignant cells were serum-starved for a period of 8 hours, and then recombinant mouse Wnt3a (100 ng/ml) was added to the medium to stimulate cells for a total 12 h period. During the last 3 hours, both SW480 and RKO cells were also incubated in the absence or presence of rottlerin (3 µM). As it can be observed in Figure 2A, whereas PKCδ inhibition did not affect the β-catenin-mediated transcriptional activity in SW480 cells, it did enhance the Wnt3a-stimulated β-catenin transcriptional activity in RKO cells. Because rottlerin has been reported to inhibit other kinases such as GSK3 and PDK1 [22], we decided to confirm these results by genetic approaches such as the expression of a dominant negative form of PKCδ. To this end, cells were transiently co-transfected with void plasmid or with 1 µg of the dominant-negative K376R PKCδ plasmid and with the TOPFlash/FOPFlash reporter. As it can be observed in Figure 2B, β-catenin/TCF transcriptional activity was increased only in RKO cells (stimulated or not with Wnt3a), expressing the PKCδ-kinase-dead mutant and was not affected in SW480 cells (Figure 2B), confirming the results obtained previously with rottlerin. In addition, when pTOPFlash- or pFOPFlash- transfected RKO cells stimulated with Wnt3a were incubated in the absence or presence of increasing amounts of rottlerin, β-catenin-mediated transcriptional activity was increased in dose-dependent fashion, until it reached a plateau at 6 µM, as it can be observed in Figure 2C. Taken together, these results suggest that PKCδ negatively modulates canonical Wnt signaling in RKO cells.

### PKCδ Knockdown Increased β-catenin- Mediated Transcriptional Activity

**T**o further confirm that the observed effects were mediated by PKCδ inhibition, we employed an RNAi approach to block PKCδ expression transiently. It is noteworthy that the plasmids used, which were obtained from Addgene, had been constructed and previously successfully probed by Dr. Alex Toker [20], who donated the plasmids to Addgene (see the Materials and Methods section). As we show in Figure 2D (upper), a 72% reduction in the PKCδ protein level was observed 24 h after transfection with 1 µg siRNA plasmid in comparison with plasmid control. Maintaining the total amount of transfected DNA unchanged (1 µg), we decreased the siRNA-PKCδ plasmid concentrations (0.7 µg, 0.5 µg, and 0.3 µg) to determine whether the effects on β-catenin reporter transcriptional activity were dose-dependent. The results presented in Figure 2D (bottom) clearly show that reporter activity increased as siRNA-PKCδ was augmented, confirming the notion that PKCδ may modulate canonical Wnt signaling in a negative manner. Interestingly, these results were obtained in cells that were not stimulated with Wnt3a; thus, they show that PKCδ knockdown leads to ligand- independent activation of the reporter.

### PKCδ Inhibition Increased the Expression of Canonical Wnt Target Genes and Cell Proliferation

**Figure 3:**
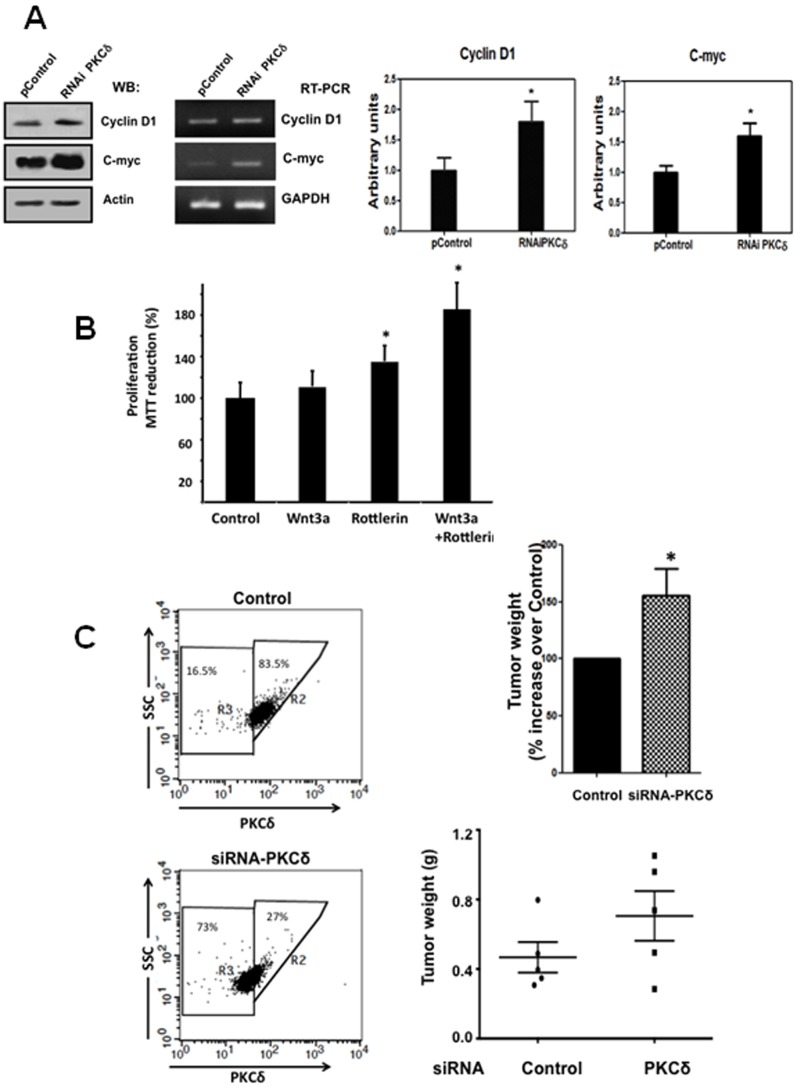
PKCδ inhibition increases Wnt3a-induced cell proliferation and the expression of Wnt target genes in RKO cells. **A)** Effect of PKCδ knockdown on Wnt target genes expression. RKO cells were transfected with 1 µg of pSuperPKCδ. RNAi or with control plasmid. 28 hours after transfection, cell lysates were prepared to perform Western blot analysis for c-myc and cyclin D1 protein expression (left) or to obtain RNA to perform RT-PCR for c-myc and cyclin D1 mRNA expression (right), respectively. The bar graphs show the quantitative scans of the ratio of c-myc or cyclin D1 protein to the actin content. Values plotted are means ± S.E.M. of at least three independent experiments. *p<0.05 **B)** Cell proliferation assay. RKO cells were serum starved (2% instead of 10%) during 8 hr and then incubated 24 hr in the absence or presence of 100 ng/ml Wnt3a and in the absence or presence of 6 µM rottlerin. The cells were then treated with 0.5 mg/ml (final concentration) of MTT solution for 3 h at 37°C. Reaction was stopped and the cell samples quantified spectrophotometrically at 570 nm. *p<0.05. **C)** Stable knockdown of PKCδ increases tumorigenic activity of engrafted RKO cells in nude mice. Stable control or PKCδ knockdown transfectants were obtained as described under Methods sections. Selected clones were picked and screened for PKCδ silencing in comparison with control cells by flow cytometry as shown at the left of the figure. Selected cells were grown (corresponding to 73% PKCδ depleted cells) and used for engraftment. 1 × 10^6^ cells per injection site were subcutaneously injected into the flanks of NOD/SCID nude mice (n = 5). Each mouse was injected at the right flank with control cells, and at the left flank with knockdown PKCδ cells. Five weeks after inoculation, animals were euthanized and tumors were removed and weighted. In all mice, engrafted PKCδ knockdown cells produced bigger tumors in comparison with the engrafted control cells injected in the same animal. The % increase in tumor weight of knockdown cells of each tumor over its respective control was calculated and depicted as the bar graph shown at the left (representing the mean weight increase over controls ± SEM obtained from 5 mice *p<0.05)**.** At the right, the same data are plotted to show the heterogeneity of the tumors.

Because canonical Wnt signaling activation promotes cell proliferation via the expression of target genes such as *C-MYC* and *CYCLIN D1*, we hypothesized that PKCδ inhibition or its knockdown in Wnt3a-stimulated RKO cells might increase the expression of Wnt target genes and cell proliferation. To test this, we examined the effect of PKCδ knockdown on the expression of *C-MYC* and *CYCLIN D1* by Western blot and PCR. Figure 3A shows that the protein levels of both c-myc and cyclin D1 significantly increased 28 h after transfection of RKO cells with the siRNA- PKCδ plasmid, in comparison with cells transfected with control plasmid. It can also be observed that only *C-MYC* mRNA levels were augmented as result of PKCδ depletion, probably because *CYCLIN D1* is transcriptionally regulated by many other signaling pathways. To examine cell proliferation, RKO cells were serum-starved (2% instead of 10%) during 8 h and then were incubated for 24 h in the absence or presence of 100 ng/ml Wnt3a and in the absence or presence of 6 µM rottlerin. After this, MTT was added to the medium for 3 h and the presence of metabolically active viable cells was used as an index of cell proliferation. As it can be observed in Figure 3, panel B, the PKCδ inhibitor increased cell proliferation *per se*, in the absence of Wnt3a, in comparison with control cells incubated without both the ligand and rottlerin. Interestingly, the addition of rottlerin to Wnt3a RKO-stimulated cells induced a nearly two-fold increase in cell proliferation with respect to control cells. Taken together, these results indicate that PKCδ negatively affects tumor cell proliferation via negative regulation of Wnt signaling pathway.

### PKCδ Silencing Affects the Tumorigenic Activity of Engrafted RKO Cells

To determine the effect of siRNA-mediated knockdown of PKCδ in tumorigenic activity of RKO cells, we made use of a xenograft model in immunocompromised mice. Stably transfected RKO cells with control pSUPER plasmid or with pSuperPKCδ-RNAi were obtained as described under Methods section, and selected by FACS analysis. As shown in Figure 3C (left), stable transfectants exhibiting more than 73% decreased expression of PKCδ were obtained. These cells were isolated, grown and subcutaneously injected into the left flanks of NOD/SCID mice. In the same animals, control cells stably transfected with pSuper control plasmid and thus expressing wild type PKCδ were subcutaneously injected into the right flanks of each animal. After five weeks, mice were killed by cervical dislocation and tumors were removed and weighted. Notably, in each engrafted mouse (n = 5), the weight of the tumor produced at the time of sacrifice by knockdown cells was clearly bigger than the weight of the tumor produced from control cells injected in the same animal (Figure 3C). Therefore, these results clearly indicated that PKCδ-depleted cells exhibit greater tumorigenic activity in grafted mice indicating an important role for PKCδ in tumor growth suppression *in vivo.*

### PKCδ Modulates Canonical Wnt Activation Participating in the Regulation of β-catenin Protein Levels Both at the Cytoplasm and the Nuclei

**Figure 4:**
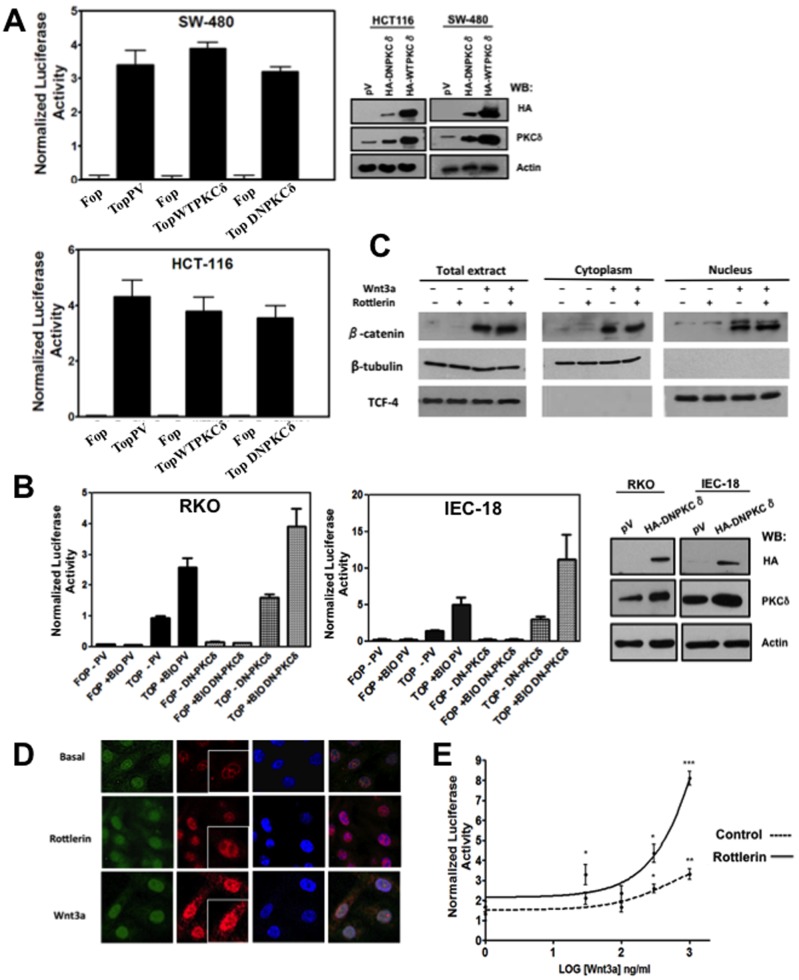
PKCδ modulates canonical Wnt activation participating in the regulation of β-catenin protein levels both at the cytoplasm and nuclei. **A)** Over-expression of wild type PKCδ or expression of a dominant-negative form of PKCδ does not affect the constitutive active β-catenin transcriptional activity of SW480 cells (expressing truncated APC) and of HCT116 cells (expressing a β-catenin degradation-resistant mutant). Cells were co-transfected with 1 µg of dominant-negative HA-tagged K376R PKCδ plasmid and with the TOPFlash/FOPFlash reporter or with 1 µg of HA- wild type PKCδ plasmid and with the TOPFlash/FOPFlash reporter. Control cells were co-transfected with void plasmids and with the reporter. After 24 h post-transfection, the reporter activity was assayed and normalized with respect to the total protein content in the cell lysate. The Western blot analysis of the expression of endogenous and transfected proteins is shown at the right of the figure. The results represent the means ± S.E.M. of at least three independent experiments and differences found were not statistically significant. B) Expression of a dominant negative form of PKCδ in non-malignant IEC-18 cells also induces an increase in β-catenin transcriptional activity. Cells were co-transfected with 1 µg of dominant-negative HA-tagged K376R PKCδ plasmid and with the TOPFlash/FOPFlash reporter Control cells were co-transfected with void plasmids (pV) and with the reporter. After 24 h post-transfection, the cells were incubated 16 h in the absence or presence of 5 µM BIO. Cells were lysed and the reporter activity was assayed and normalized with respect to the total protein content in the cell lysate. The Western blot analysis of the expression of endogenous and HA-transfected PKCδ is shown at the right of the figure. The results represent the means ± S.E.M. of at least three independent experiments. C) PKCδ inhibition induces β-catenin protein stabilization. RKO cells were incubated in the absence or presence of Wnt 3a conditioned medium for a total 8 h period and in the absence or presence of rottlerin (6 µM) during the last 3 hours. Cytoplasmic and nuclear fractions were then obtained from the cell extracts employing a nuclear isolation kit (Sigma) and the presence of β-catenin analyzed in each fraction by Western blot. β-tubulin was employed as cytoplasmic marker and the transcription factor TCF4 as nuclear marker to confirm no contamination and thus, successful fractionation. The data are representative of three independent experiments. D) The PKCδ inhibition increased β-catenin immunoreactive signal particularly at the cell nuclei. RKO cells were incubated in the absence (basal) or presence of 6 µM rottlerin for 3 h, and in the absence or presence of 100 ng/ml Wnt3a for 6 h. Cells were fixed, permeabilized and coimmunostained with antibodies against PKCδ and β-catenin. Fluorescence was analyzed by laser confocal microscopy as described under “Materials and Methods”. PKCδ was visualized with FITC-conjugated goat anti-rabbit antibody and β-catenin with TRITC-conjugated goat anti-mouse antibody. Data are representative of four independent experiments. E) PKCδ inhibitor increases the transcriptional activity of β-catenin induced dose-dependently by Wnt3a. RKO cells were transfected with TOPFlash reporter plasmid. 24 h post-transfection, cells were serum starved for 8 h and incubated in the absence or presence of increasing concentrations of Wnt3a for a total 8 h period. During the last 3 h, cells were also incubated in the absence or presence of rottlerin 6 µM. Then, cells were washed, homogenized and luciferase activity was measured and normalized with respect to the total protein content in the cell lysate. The results represent the means ± S.E.M. of three independent experiments. *p<0.05, **p<0.01, ***p<0.001.

Potentiation of β-catenin transcriptional activity upon PKCδ activity blockade was observed in RKO cells that express wild-type APC, but not in SW480 cells that express mutant truncated APC. In order to investigate whether the expression of normal APC is necessary to produce the PKCδ inhibition effect, we made use of the colon carcinoma cell line HCT116. These cells express wild-type APC but also express a β-catenin degradation-resistant mutant (S33Y); thus, like SW480 cells, they possess constitutively active β-catenin transcriptional activity**.** In addition, we also examined in these cells whether a gain- of- function experiment overexpressing PKCδ, whose expression is diminished in colon malignant in comparison with non- malignant cells (see Figure1A), could repress transcriptional activity. HCT116 and SW480 **c**ells were co-transfected with 1 µg of dominant-negative, HA-tagged K376R PKCδ plasmid and with the TOPFlash/FOPFlash reporter or with 1 µg of HA- wild-type PKCδ plasmid and with the TOPFlash/FOPFlash reporter. Control cells were co-transfected with void plasmids (pV) and with the reporter. Luciferase activity was examined at 24 h post-transfection, as shown in Figure 4A. The expression level of endogenous, HA-kinase-dead or HA-wild-type PKCδ is depicted at the right of the figure. As it can be observed, β-catenin transcriptional activity was neither affected in SW480 nor in HCT116 cells expressing a dominant-negative form of PKCδ or a wild-type PKCδ, since the small differences observed were not statistically significant. Thus, these results indicated that the expression of normal APC does not explain the PKCδ-mediated effects and that PKCδ overexpression in these cells does not rescue the negative effects observed in β-catenin transcriptional activity as a result of PKCδ inhibition. The possibility that a further increase in PKCδ activity blockade-induced reporter activity would be masked by the very robust, constitutively active β-catenin transcriptional activity observed in both SW480 and HCT116 cells was ruled out, because PKCδ inhibition did not also increase β-catenin-mediated transcriptional activity in the HT-29 cell line, which express only truncated forms of APC but show low basal β-catenin transcriptional activity (data not shown).

In view of these results, we reasoned that all cells in which PKCδ inhibition failed to improve β-catenin transcriptional activity have a non-functional β-catenin degradation complex in common. Taking into account that APC plays a crucial role in regulating β-catenin stability in this complex, and that it has been reported that the affinity of APC for several proteins in this complex is controlled by phosphorylation, we hypothesized that PKCδ may negatively impinge upon the canonical Wnt signaling participating in the control of β-catenin stability via its interaction with APC. According to this hypothesis, the inhibition of PKCδ activity in normal non malignant intestinal cells (with normal β-catenin degradation complex) would produced the same effects observed in malignant RKO cells. To investigate this, we made use of the rat intestinal epithelial cell line IEC-18 because 112 CoN human cells are very hard to transfect, and we used a selective inhibitor of GSK3β (“BIO”) to stimulate canonical Wnt signaling in these cells. IEC-18 and RKO **c**ells were co-transfected with 1 µg of dominant-negative, HA-tagged K376R PKCδ plasmid and with the TOPFlash/FOPFlash reporter. Control cells were co-transfected with void plasmids (pV) and with the reporter. Then, **c**ells were incubated 16 h in the absence or presence of the GSK3β inhibitor BIO (5 µM) and luciferase activity was examined, as shown in Figure 4B. The expression level of endogenous and HA-kinase-dead PKCδ is depicted at the right of the figure. As it can be seen, β-catenin/TCF transcriptional activity was increased both in RKO and in IEC-18 cells as result of GSK3β inhibition with BIO in comparison with control, non- treated cells. Interestingly, the expression of a dominant negative form of PKCδ also increased *per se* the transcriptional activity in the absence of BIO in both cell types, and remarkably, the expression of the PKCδ-kinase-dead mutant produced an additive effect with BIO to stimulate β-catenin/TCF transcriptional activity in both RKO and IEC-18 cells. Thus, these results confirmed the notion that PKCδ may modulate canonical Wnt signaling in a negative manner whenever exists a functional β-catenin degradation complex.

To determine the effects of PKCδ inhibition on β-catenin protein levels, RKO cells were incubated in the absence or presence of Wnt3a conditioned medium for a total 8 h period and in the absence or presence of rottlerin (6 µM) during the last 3 h. Cytoplasmic and nuclear fractions were then obtained from the cell extracts and the presence of β-catenin was analyzed in each fraction by Western blot. The fractionation was successful and clean, as shown by the presence of β-tubulin in total cell extract and cytoplasm but not at nuclei, and by the presence of the transcription factor TCF4 at cell extract and nuclei but not at the cytoplasm. The results presented in Figure 4C indicated that β-catenin protein levels were increased because of Wnt3a treatment of cells, as expected, but interestingly, these levels were also slightly increased by rottlerin alone at the cytoplasm and at the nuclear fraction, suggesting that PKCδ inhibition favors β-catenin stabilization and its translocation to the nuclear compartment. It can also be observed that β-catenin at the nucleus appeared as two bands that were both increased as the result of PKCδ inhibition. Consistent with these results, the immunofluorescence assays followed by confocal microscopy analysis presented in Figure 4D, showed that RKO cells that were incubated in the absence (basal) or the presence of 6 µM of rottlerin for 3 h exhibited an increase in the immunoreactive signal corresponding to β-catenin at the nuclei (zoom-in), but as expected, Wnt 3a treatment of cells induced a more prominent β-catenin stabilization and nuclear accumulation. In addition, when the pTOPFlash-transfected RKO cells were incubated in the absence or presence of 6 µM of rottlerin and in the absence or presence of increasing amounts of Wnt3a, β-catenin transcriptional activity was increased in a dose-dependent manner by Wnt3a, but in the presence of rottlerin, this was higher, even at the basal point obtained without ligand (Figure 4E). Taken together, these results suggest that PKCδ negatively modulates the canonical Wnt pathway, probably acting at the β-catenin degradation complex.

**Figure 5:**
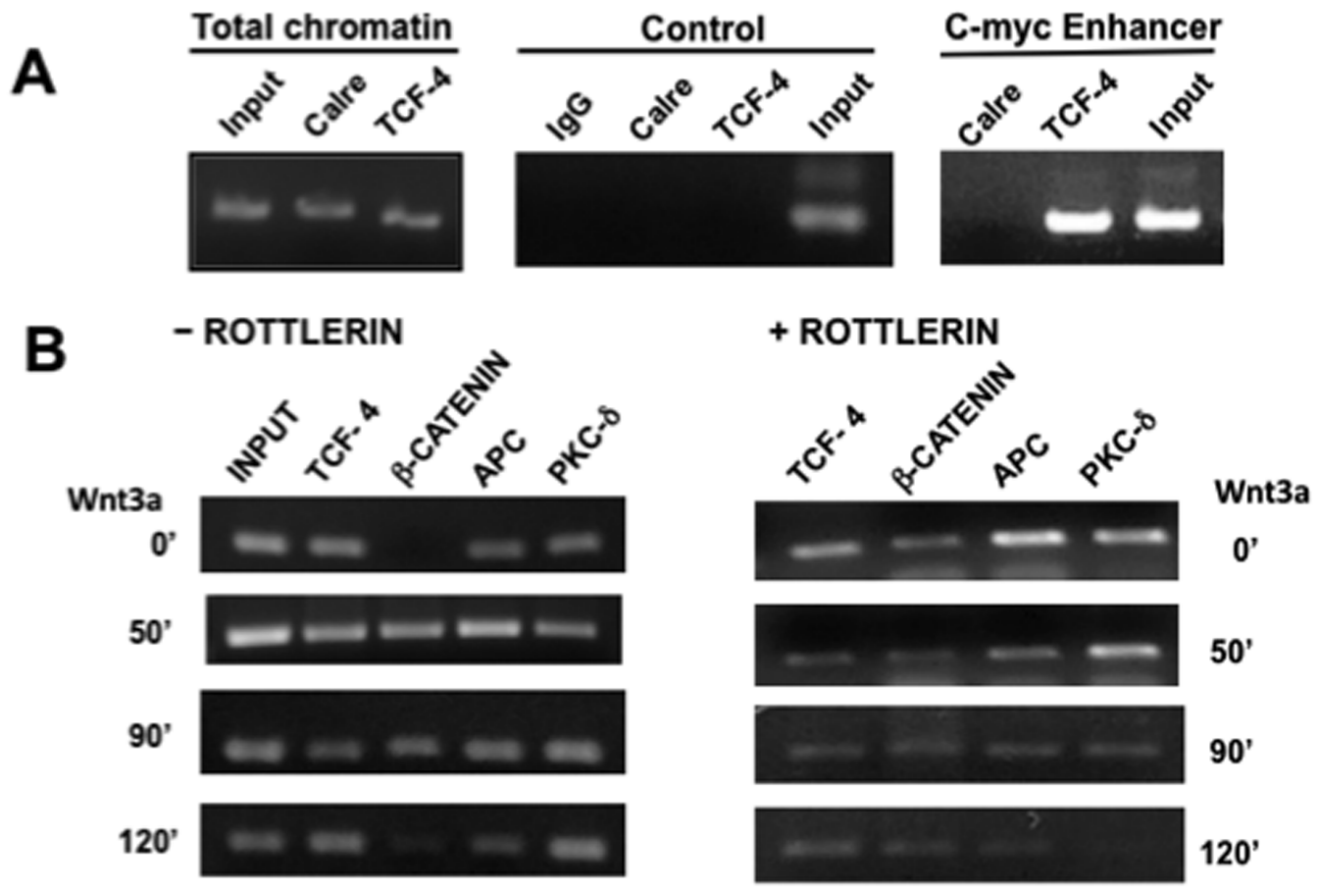
Both PKCδ and APC bind to the c-Myc enhancer in vivo. Serum starved RKO cells (8 h) were pre-incubated for 2 h in the absence or presence of the PKCδ inhibitor rottlerin (6 µM), and then incubated in the absence or presence of Wnt3a conditioned medium during 50, 90 or 120 min. The ChIP analysis of the time course of cell treatment was performed as described in Materials and Methods section. PCRs were done with primers spanning the upstream TCF/LEF sites in the human *C-MYC* gene enhancer (spanning the region corresponding to −1447 to −1144 relative to the P1 RNA start site +1) as described in (9). PCR of the input DNA prior to immunoprecipitation was used as a control. **A)** ChIP specificity controls: conditions employed were specific, because TCF4 and calreticulin bound to total chromatin, but calreticulin, a protein not related to Wnt signaling did not bind to the c-Myc enhancer but the TCF4 transcription factor appeared bound to it. Neither calreticulin nor TCF4 were found bound at a distal upstream region (1 kb) of the *C-MYC* gene (control). **B)** Time course of RKO cells treatment in the absence or presence of Wnt3a conditioned medium and in the absence or presence of rottlerin. Results shown are representative of three experiments using different cell preparations.

Because APC was detected also co-localizing with PKCδ at the nucleus, we then asked whether they are present at Wnt target gene promoters *in vivo*. To examine this, we carried out ChIP analyses of the *C-MYC* enhancer of RKO cells preincubated for 2 h in the absence or presence of the PKCδ inhibitor rottlerin, and then incubated in the absence or presence of Wnt3a conditioned medium during several periods. ChIP analysis of the cell treatment’s time course is shown in Figure 5. The ChIP conditions employed here were specific, because only TCF4, and not calreticulin, which is a protein not related to Wnt signaling, appeared bound to the *C-MYC* enhancer, as shown in the figure (panel A, top), and also because TCF4 was not detected as bound at a distal upstream region of the *C-MYC* gene (control, panel A). As can be observed, TCF4 was found constitutively bound to the *C-MYC* enhancer as expected. In contrast, β-catenin was not bound to the enhancer in the absence of both Wnt3a ligand and in the absence of rottlerin (time 0), but was recruited to the enhancer upon Wnt3a treatment and, interestingly, also in response to rottlerin alone (time 0). In addition, the results clearly demonstrated that both PKCδ and APC are present at the *C-MYC* enhancer in the absence or presence of rottlerin, but their binding to the enhancer diminishes after 120 min of rottlerin treatment. Taken together, these results are consistent with the notion that PKCδ inhibition promotes β-catenin stabilization and translocation to the nucleus, and indicate that PKCδ was present at the *C-MYC* enhancer *in vivo* in RKO cells, suggesting that it may function in the nucleus as a transcriptional modulator of Wnt target gene expression.

### APC is Phosphorylated by PKCδ in Intact RKO Cells

**Figure 6:**
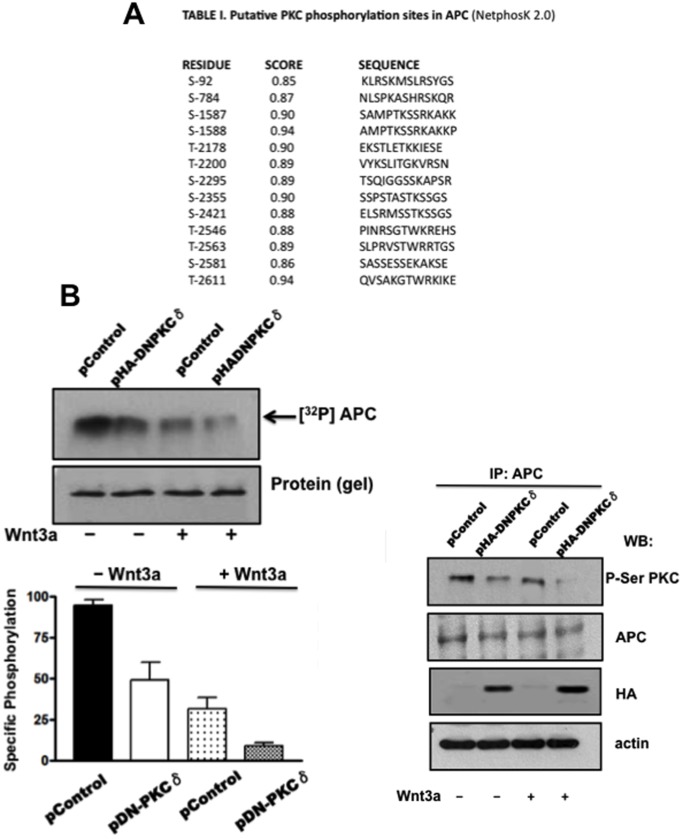
PKCδ phosphorylates APC in intact RKO cells. **A)** Table I. Putative PKC phosphorylation sites in APC (NetphosK 2.0). **B)** Metabolic labeling and determination of specific phosphorylation (left). Western blot with anti Phospho-Ser-PKC substrate (right). 24 h post-transfection of RKO cells with the pControl (void) or pHA-DNPKCδ plasmids, RKO cells were serum-starved (2% instead of 10%) for 4 h, washed and incubated with 150 µCi/ml [^32^P] Pi in phosphate-free DMEM medium supplemented with 2% FBS for a total period of 4 h. Wnt3a (100 ng/ml, or vehicle) was added during the last hour of incubation period as described in Material and Methods. APC was immunoprecipitated from the corresponding cell lysates, subjected to SDS-PAGE, transferred to nitrocellulose, and processed for autoradiography or for Western blotting. Autoradiography: the determination of protein content was done after radiography in the corresponded dried gel. Autoradiograms and their corresponding dried gels were quantified with an image densitometer and the specific phosphorylation was determined as the ratio of phosphorylated protein to the total protein content. Values plotted are means ± S.E.M. for three experiments performed with different cell preparations. Western blotting: APC immunoprecipitates were analyzed for the presence of APC phosphorylated in Ser residues by PKC. The same blots were erased and reprobed with APC antibodies. Also is shown the HA-tagged kinase-dead PKCδ over-expression and actin loading controls. A representative experiment from three experiments performed with different cell preparations is shown.

Given that it has been demonstrated that phosphorylation is an important mechanism of APC regulation for modulating its affinity for β-catenin, we examined whether APC could be a PKCδ substrate. An *in silico* analysis of the aminoacid APC sequence utilizing the NetphosK 2.0 program (http://expasy.com/) showed that APC has many high-score putative PKC phosphorylation sites distributed throughout the protein (Figure 6A, Table 1), and interestingly, some of these are located at positions involved in Wnt regulation by APC, including the 15 amino-acid β-catenin-binding repeats, the 20 amino-acid repeats, particularly R2, which has been recently demonstrated to play a crucial role for APC function in the β-catenin destruction complex [23], and the SAMP repeats that bind axin. To examine whether PKCδ phosphorylates APC in intact RKO cells, 24 h post-transfection with the pHACE (void) or pDN-PKCδ plasmids, RKO cells were serum-starved (2% instead of 10%) for 4 h, washed and incubated with 150 µCi/ml [^32^P] Pi in phosphate-free DMEM medium supplemented with 2% FBS for a total period of 4 h. During the last hour, cells were also incubated in the absence or presence of Wnt3a (50 ng/ml). Cells lysates from control and treated cells were immunoprecipitated with antibodies directed against APC and resolved by SDS-PAGE (5−15%-gradient); the gels were subjected to autoradiography or were transferred to nitrocellulose membranes for Western blot analysis. We employed an antibody specific for detection of phospho-serine-PKC substrates (Cell Signaling). The results shown in Figure 6B demonstrated that the basal phosphorylation of APC, either analyzed by autoradiography or by Western blotting, was decreased more than 50% in cells expressing a dominant negative form of PKCδ (pControl without Wnt3a). Interestingly, treatment of cells with Wnt3a induced *per se* a decrease in APC phosphorylation, but this decrease was even more drastic in cells expressing the dominant negative PKCδ. These results indicated that PKCδ mediates the phosphorylation of APC *in vivo*, and suggest that APC function might be regulated by PKCδ phosphorylation.

**Table 1:** Putative PKA phosphorylaltion sites in APC (NetphosK 2.0 http://expasy.com/).

RESIDUE	SCORE	SEQUENCE
S-92	0.85	KLRSKMSLRSYGS
S-784	0.87	NLSPKASHRSKQR
S-1587	0.90	SAMPTKSSRKAKK
S-1588	0.94	AMPTKSSRKAKKP
T-2178	0.90	EKSTLETKKIESE
T-2200	0.89	VYKSLITGKVRSN
S-2295	0.89	TSQIGGSSKAPSR
S-2355	0.90	SSPSTASTKSSGS
S-2421	0.88	ELSRMSSTKSSGS
T-2546	0.88	PINRSGTWKREHS
T-2563	0.89	SLPRVSTWRRTGS
S-2581	0.86	SASSESSEKAKSE
T-2611	0.94	QVSAKGTWRKIKE

## Discussion

The process of intestinal carcinogenesis is associated with alterations in the activity and expression of Protein kinase C (PKC) isoforms. On the other hand, Wnt signaling has been well characterized as one of the most important contributors to tumorigenesis, and it has been implicated in many types of solid tumors. Although many studies have implicated PKC isoforms in Wnt signaling, particularly in non-canonical Wnt activation, the molecular mechanisms involved in their crosstalk remain poorly understood. In this study, we report that PKCδ negatively modulates canonical Wnt signaling participating in the control of β-catenin protein levels.

PKCδ has been widely implicated as a mediator of apoptosis in response to phorbol esters and chemotherapeutic agents, and it is differentially expressed in several human cancer types. In the intestinal epithelium, PKCδ has been reported to function as a tumor suppressor [15]–[17]. Here we have shown that despite that PKCδ expression is diminished in malignant in comparison with non-malignant cells, PKCδ selectively co-immunoprecipitates with the tumor suppressor APC, both with wild-type expressed in normal and in RKO cancer cells, as well as with truncated versions of APC expressed in HT-29 and SW480 malignant cells. The interaction is specific, because neither PKCε (Figure 1C) nor PKCβII (not shown), which are overexpressed in malignant cells, co-immunoprecipitated with APC. In addition, our results show that co-localization of APC with PKCδ occurs at the cytoplasm and at the cell nucleus both in normal and in malignant cells (Figure 1D). These data suggest that their interaction may be physiologically relevant for APC functions and that the amino-terminal half of APC is probably involved in this interaction.

We have found that the pharmacological inhibition of PKCδ, expression of a dominant-negative PKCδ, or its knockdown by siRNA in cancerous RKO cells, improved in a dose-dependent way the β-catenin transcriptional activity, the expression of Wnt target genes (*CYCLIN D1* and *C-MYC*) and Wnt3a-induced proliferation of cells. However, we have observed that these effects are solely induced in cells that possess a functional β-catenin degradation complex. Consistent with this, the expression of a dominant negative form of PKCδ in non- malignant IEC-18 cells reproduced the effects seen in RKO cells (Figure 4B). In addition, we have found that PKCδ is involved in APC phosphorylation *in vivo*. Our data are consistent with a model in which PKCδ negatively modulates canonical Wnt signaling by a molecular mechanism involving the relief of the negative influence of APC on β-catenin levels, probably by PKC-mediated APC phosphorylation. This phosphorylation of APC would favor its function at the β-catenin degradation complex, resulting in decreased β-catenin levels.

It is well established that phosphorylation is a crucial mechanism for regulating all known APC functions [24]–[26]. However, it remains unknown, which kinase phosphorylates which APC site *in vivo*. APC is a phosphoprotein that can be phosphorylated by many kinases, including GSK3β, Mitogen-activated protein kinase (MAPK), cyclin-dependent kinases, protein kinase A (PKA), casein kinase I and II (Ck1 and Ck2) and calmodulin kinase [27]–[29]. Importantly, it also has many putative phosphorylation sites for PKC distributed throughout the protein, and with a high probability score, as shown here (Table I in figure 6). *In vivo* and *in vitro* studies have supported the hypothesis that the 20 amino acid repeats are phosphorylated and that this is important for the interaction of APC with β-catenin [29], [30]. Indeed, the analysis of the crystal structure of a complex between the armadillo repeats 1–5 of β-catenin and a phosphorylated APC 20 amino acid repeat region revealed that phosphorylation enhances their interaction by 300- to 500-fold [25]).

With respect to the role of phosphorylation in APC functions at the degradation complex, so far, several models of the inner functioning of the destruction complex have been proposed to date, each suggesting different mechanistic roles for APC in targeting ß-catenin for destruction. All proposed models take into account the complex structure of APC. The third APC N-terminal includes a block of armadillo repeats that bind multiple partners, with the full repertoire yet to be defined. The middle third of APC carries a series of short binding sites for proteins involved in Wnt regulation, including 15- and 20-amino acid repeats (15R and 20R), which bind ß-catenin and SAMP repeats, which bind Axin. It also contains the short conserved sequence B [31], also known as the Catenin inhibitory domain (CID) [32]. Proposed models also consider the fact that both APC and Axin have ß-catenin binding sites, but unlike APC, Axin has a single ß-catenin binding site. Under basal conditions, ß-catenin’s affinity for Axin is higher than that for APC. APC, however, is a casein kinase 1 (CK1) and GSK3 substrate, with phosphorylation sites within the 20Rs [33]. Phosphorylated APC possesses higher affinity for ß-catenin than for Axin [24], [25], [34]. This led several authors [23], [24], [35] to propose that the destruction complex undergoes a cycle of structural rearrangements. It assembles with Axin bound to ß-catenin due to its higher affinity. ß-catenin and APC are both phosphorylated by GSK3, triggering the transfer of ß-catenin to APC. This is suggested to facilitate ß-catenin transfer to the E3 ubiquitin ligase, with presumed APC dephosphorylation by PP2A resetting the system. Interestingly, there are several PKC putative phosphorylation sites in APC at R1, R2, R6 and R7 amino acid repeats, as depicted in Figure 6, particularly if it is taken into account that R2, which has been highly conserved throughout evolutionary time, has recently been reported as essential for targeting ß-catenin for destruction [23].

With respect to the nuclear functions of APC, it is well known that APC is a nucleocytoplasmic shuttling protein: two functional nuclear localization signals and four nuclear export signals have been identified within APC. This shuttling plays an important role in APC’s tumor suppressor function because it serves to promote nuclear export of ß-catenin [36]. Nuclear APC also directly counteracts ß-catenin activation at Wnt target genes and is necessary for the periodic cycling of co-activator and co-repressor complexes at Wnt enhancers [9].

Post-translational modifications govern the interactions among different Wnt transcriptional regulators. Phosphorylation has also been shown to play an important role in regulating APC activity. In this respect, it has been demonstrated that the 20 phosphorylated amino acid repeats of APC, but not the unphosphorylated ones, effectively disrupt TCF binding to ß-catenin [25]. In addition, it has been reported that phosphorylation of APC by CK1 *in vitro* dramatically enhances its affinity for ß-catenin [24], [25], which would disfavor binding to LEF-1/TCF. ChIP studies indicate that LEF-1 binds constitutively to Wnt enhancers, whereas the co-activator and co-repressor proteins alternate on and off the DNA during active transcription [9]. This cycling of enhancer components appears to be triggered by post-translational modifications, especially ubiquitination.

In view of the results presented here, one attractive possibility is that the affinity of APC for several proteins in the promoter or enhancer complex, as well as in the destruction complex, is controlled by post-translational protein modifications such as PKCδ-mediated phosphorylation. Thus, phosphorylation of APC by PKC would induce it to bind tightly to ß-catenin and to prevent the latter from binding to the destruction complex at the cytoplasm and/or to TCF4 at the cell nuclei. It remains to be elucidated which proteins, in addition to APC, can be phosphorylated by PKCδ at Wnt target gene promoters in order to participate in its transcriptional repression.

## Supporting Information

Figure S1 Kinase activity was measured with the PKCδ immune complexes obtained from partly purified and concentrated PKC (1 mg**/**ml) obtained from rat hepatocytes. Kinase activity was initiated by resuspending the immunoprecipitates in 50 µl of assay mixture, consisting of kinase buffer [20 mM Tris**/**HCl (pH 7.5)**/**10 mM MgCl**_2_/**0.5 mM CaCl**_2_/**50 mM 2-mercaptoethanol] plus 20 µg/ml phosphatidylserine, 0.8 µg/ml 1,2-diolein, 10 **µ**M [**γ-** ^32^P] ATP (6000 Ci**/**mmol) and in the absence or presence of substrate added (200 µg/ml Histone H1-IIIS) to the immunoprecipitates and in the absence or presence of the indicated rottlerin concentrations. Reactions proceeded for 20 min at 30**°**C, then were terminated by the addition of 50 µl of SDS**/**PAGE sample buffer, boiled for 5 min and analyzed by SDS**/**PAGE [12.5% (w**/**v) gel] and autoradiography. The arrow indicates the position of 31 kDa phosphorylated histone H1. The positions of molecular mass markers are indicated (in kDa) at the left.
(TIFF)
